# Interaction of Variable Bacterial Outer Membrane Lipoproteins with Brain Endothelium

**DOI:** 10.1371/journal.pone.0013257

**Published:** 2010-10-22

**Authors:** Gaurav Gandhi, Diana Londoño, Christine R. Whetstine, Nilay Sethi, Kwang S. Kim, Wolfram R. Zückert, Diego Cadavid

**Affiliations:** 1 Center for Immunology and Inflammatory Diseases, Massachusetts General Hospital, Boston, Massachusetts, United States of America; 2 Department of Neurology and Neuroscience and Center for the Study of Emerging Pathogens at UMDNJ-New Jersey Medical School, Newark, New Jersey, United States of America; 3 Department of Microbiology, Molecular Genetics and Immunology, University of Kansas Medical Center, Kansas City, Kansas, United States of America; 4 Pediatric Infectious Diseases, Johns Hopkins University School of Medicine, Baltimore, Maryland, United States of America; Columbia University, United States of America

## Abstract

**Background:**

Previously we reported that the variable outer membrane lipoprotein Vsp1 from the relapsing fever spirochete *Borrelia turicatae* disseminates from blood to brain better than the closely related Vsp2 [1]. Here we studied the interaction between Vsp1 and Vsp2 with brain endothelium in more detail.

**Methodology/Principal Findings:**

We compared Vsp1 to Vsp2 using human brain microvascular endothelial cell (HBMEC) association assays with aminoacid radiolabeled Vsp-expressing clones of recombinant *Borrelia burgdorferi* and lanthanide-labeled purified lipidated Vsp1 (LVsp1) and Vsp2 (LVsp2) and inoculations of the lanthanide-labeled proteins into mice. The results showed that heterologous expression of LVsp1 or LVsp2 in *B. burgdorferi* increased its association with HBMEC to a similar degree. Purified lanthanide-labeled lipidated Vsp1 (LVsp1) and LVsp2 by themselves were capable of associating with HBMEC. The association of LVsp1 with brain endothelium was time-dependent, saturable, and required the lipidation. The association of Vsp1 with HBMEC was inhibited by incubation at lower temperature or with excess unlabeled LVsp1 or LVsp2 but not with excess rVsp1 or mouse albumin or an anti Vsp1 monoclonal antibody. The association of LVsp2 with HBMEC and its movement from blood to brain parenchyma significantly increased in the presence of LVsp1.

**Conclusions/Significance:**

Variable bacterial outer membrane lipoproteins interact with brain endothelium differently; the lipidation and variable features at the protein dome region are key modulators of this interaction.

## Introduction

Little is known about the interaction of bacterial lipoproteins with brain endothelium. Previous studies in our laboratory with the relapsing fever (RF) spirochete *Borrelia turicatae (Bt)* have shown that isogenic serotypes expressing different variable outer membrane lipoproteins vary in their localization in vivo: serotype 1 (Bt1), defined by expression of Variable small protein 1 (Vsp1), infects and inflames the brain better than isogenic serotype 2 (Bt2), defined by expression of Vsp2; conversely, Bt2 causes higher peak bacteremia and systemic disease than Bt1 [2]–[9]. In recent experiments using lanthanide-labeled purified lipidated Vsp1 and Vsp2 we showed that LVsp1 disseminates to and inflames the brain better than Vsp2 [1]. This same study showed that co-administration with LVsp2 displaced LVsp1 away from the brain parenchyma into brain capillaries [1].

The underlying mechanism explaining the greater ability of Vsp1 to move to the brain from the periphery remains to be determined. One possibility is that Vsp1 binds to brain endothelial cells better than Vsp2. Another possibility is that LVsp1 may be internalized and transported through brain endothelial cells better than LVsp2. Previously we observed by immunofluorescence microscopy that LVsp1 released from Bt1 can be internalized into human brain microvascular endothelial cells (HBMEC) [10]. Here we studied the interaction between Vsp1 and Vsp2 with brain endothelium using cell association assays with radiolabeled recombinant *Borrelia burgdorferi* transformants displaying LVsp1 or LVsp2 in their surface, radiolabeled sonicated proteins from Bt1, and lanthanide-labeled purified LVsp1 and LVsp2 present alone or in combination in vitro and in vivo. The results revealed that LVsp1 and LVsp2 by themselves associate with brain endothelial cells to similar degree and suggest that the ability of Vsp1 to disseminate to the brain is determined by greater ability of Vsp1 to traffic across endothelial cells into the brain parenchyma. Almost as important was the finding that the presence of Vsp1 enhances the ability of Vsp2 to cross the blood-brain barrier.

## Results

### Association of Vsp-expressing *B. burgdorferi* with human eukaryotic cells

**Figure 1:**
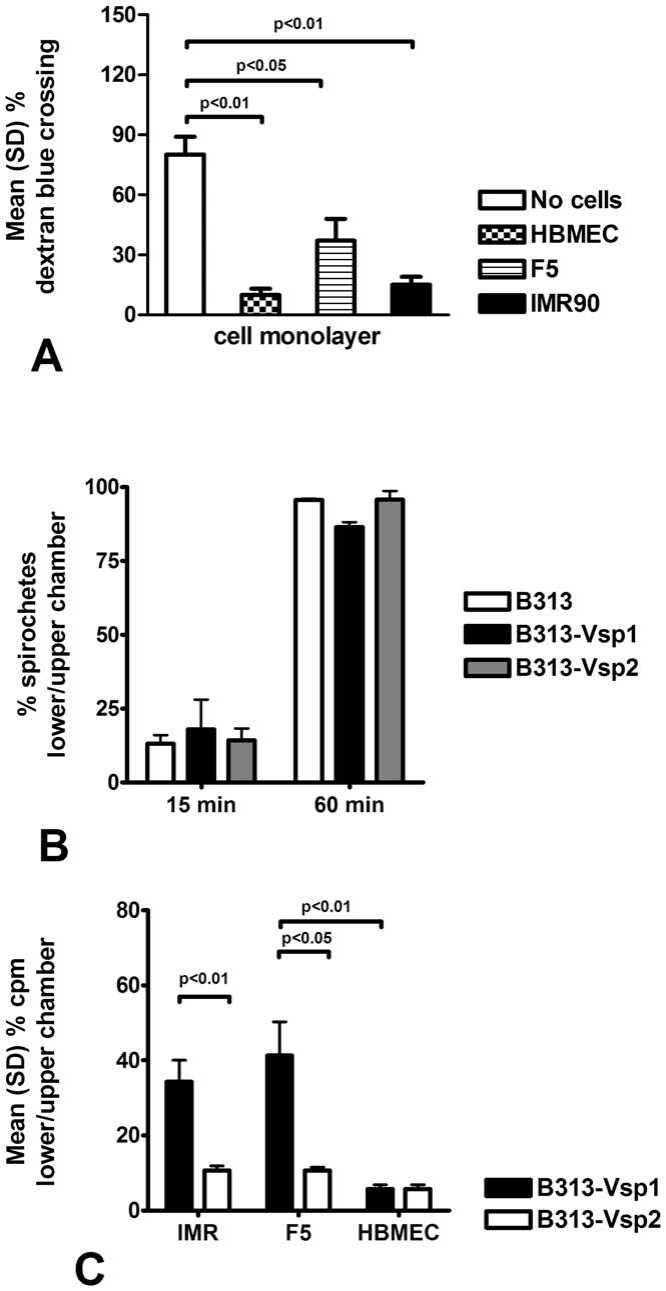
Movement of non-infectious Vsp-expressing *Borrelia burgdorferi* across eukaryotic cell monolayers. (**A**) Movement of 2000 dextran blue across confluent monolayers of human brain microvascular endothelial cells (HBMEC), human fibroblast cells (IMR90), and human arachnoidal cells (F5) compared with collagen-coated inserts alone (without cells); notice that all monolayers significantly limited the movement of 2000 dextran blue from the upper to the lower transwell chamber. (**B**) Movement of wild type (B313) and Vsp1 (B313-Vsp1) or Vsp2-(B313-Vsp2) transformants of non-infectious high passage *B. burgdorferi* B313 clones across collagen-coated inserts without cells; notice their similar movement into the lower chamber. (**C**) Movement of S35-radiolabeled B313-Vsp1 and B313-Vsp2 transformant clones across fibroblast (IMR), arachnoidal (F5), and brain microvascular endothelial cell (HBMEC) monolayers. Notice that only HBMEC severely limited the movement of both Vsp-recombinant *B. burgdorferi* transformants into the lower chamber. All experiments represent data from 3–4 transwells and were repeated at least twice for consistency.

We began this study by measuring the association of Vsp-recombinant *B. burgdorferi* with different human eukaryotic cells. First we used a high passage, non-infectious B313 strain of *B. burgdorferi* that had been previously genetically modified to express Vsp1 or Vsp2 of *B. turicatae* [11] to assess the effect of heterologous expression of either Vsp1 or Vsp2 on the association with different human eukaryotic cells. For this we selected SV-40 transformed human brain microvascular endothelial cells (HBMEC), IMR90 fibroblasts, and F5 arachnoidal cells derived from a human meningioma [10]. We compared the association of wild type and Vsp-recombinant clones of B313 with the 3 eukaryotic cells using 12 mm collagen-coated transwell chambers with confluent monolayers grown on polycarbonate membranes as before [10]. We verified that the confluent monolayers formed a physical barrier to different degree: HBMEC restricted the movement of 2000 dextran blue into the lower chamber to the highest degree (Figure 1A). Collagen-coated polycarbonate inserts without the monolayers did not restrict the movement of either Vsp1 or Vsp2-recombinant B313 (Figure 1B). This was important because *B. burgdorferi* can interact with collagen [12] and because the HBMEC culture medium contains heparin, which is bound by Vsp2 but not by Vsp1 [13]. To compare the association of Vsp-expressing recombinant B313 cells with the eukaryotic cells monolayers we inoculated motile borrelias that had metabolically labeled with S^35^-amino acids into the upper transwell chamber and measured the radioactivity in the lower chamber 4 h later. The results showed that confluent monolayers of HBMEC severely restricted the movement of both Vsp1 and Vsp2-expressing *B. burgdorferi* transformants (Figure 1C). A similar, albeit less pronounced, restriction of movement into the lower chamber was observed for B313-Vsp2 with IMR90 and F5 monolayers (Figure 1C). We concluded that Vsp1-expressing B313 cells moved better across IMR and F5 monolayers than if Vsp2 is expressed. Only HBMEC monolayers severely restricted the movement of Vsp1-B313 into the lower chamber.

### Surface display of Vsp's increases the association of *B. burgdorferi* cells with HBMEC

**Figure 2:**
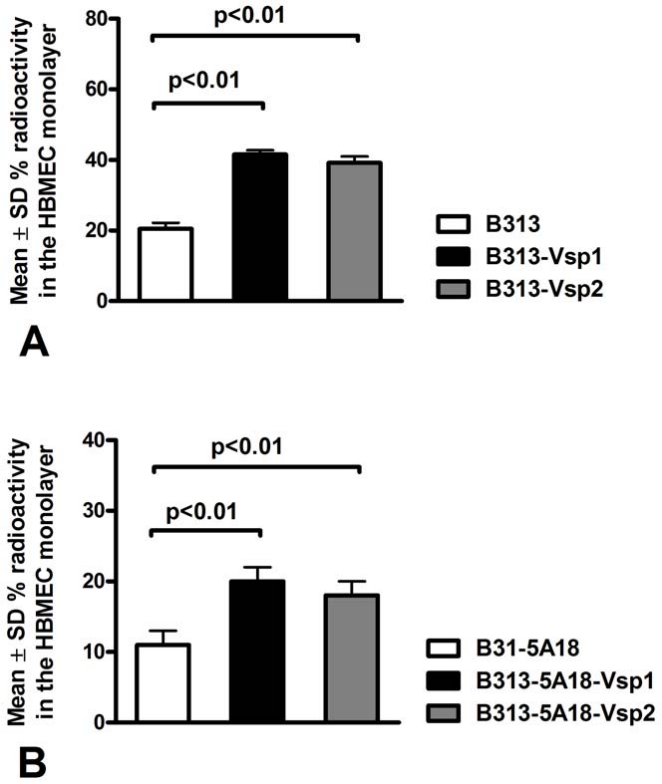
Binding of Vsp-recombinants of *Borrelia burgdorferi to human brain endothelium*. Metabolically labeled wild type or Vsp-recombinant high passage non-infectious (panel A) or low passage infectious (panel B) *B. burgdorferi* were incubated with human brain microvascular endothelial cell (HBMEC) monolayers. Binding to HBMEC monolayers was measured in transwell chambers 4–5 hours after inoculation into the upper chamber with S35-aminoacid labeled motile spirochetes. All experiments were repeated at least twice and with 3–4 transwells per borrelia. Notice significantly higher percentage of radioactivity in the HBMEC monolayer of transwell chambers inoculated with either Vsp1 or Vsp2-recombinant clones compared to wild type *B. burgdorferi*. Also notice that association with HBMEC is higher in the high passage non-infectious background (panel A).

**Figure 3:**
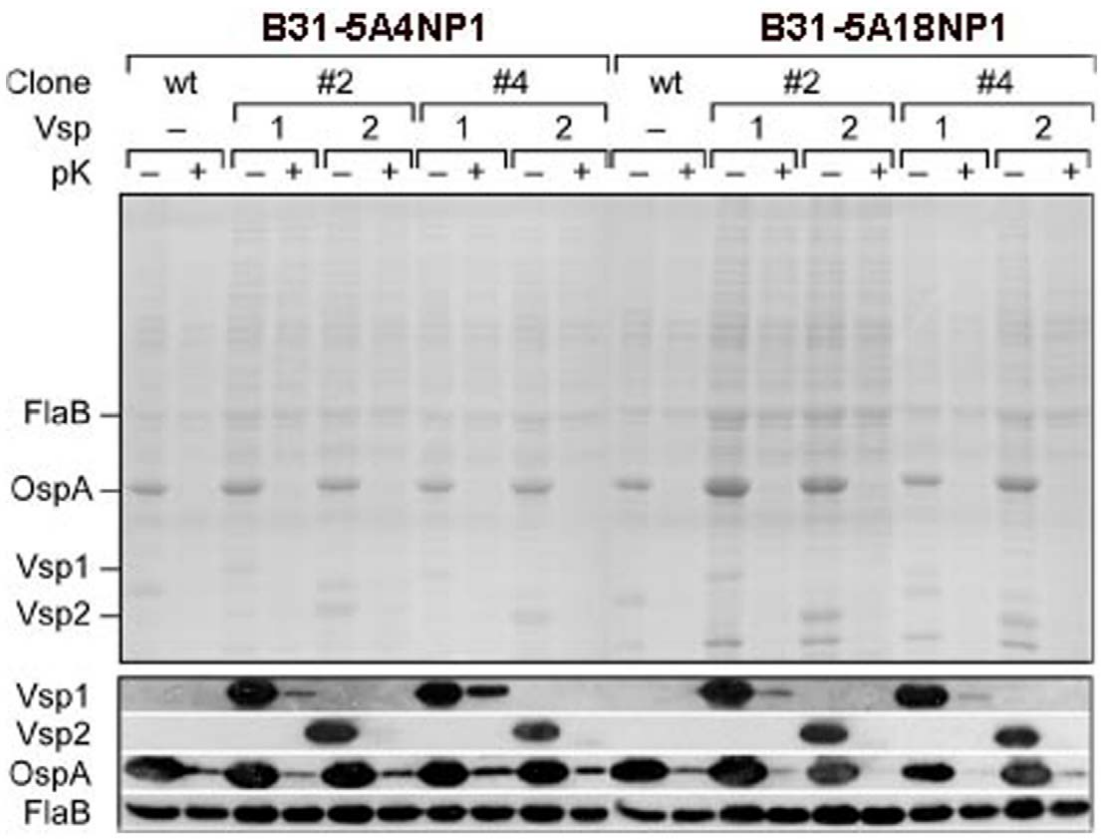
SDS-PAGE and Western blot of Vsp1- and Vsp2- expressing recombinant low passage infectious clones of *B. burgdorferi* B31. Whole-cell lysates of Vsp1 and Vsp2 recombinant clones of low passage infectious *B. burgdorferi* B31 were characterized by Coomassie-stained 12% SDS-PAGE (upper panel) and immunoblot with monoclonal antibodies to Vsp1, Vsp2, OspA and FlaB. Borrelia cells were treated (+) or not (−) in situ with proteinase K for digestion of surface-exposed proteins prior to lysis and SDS-PAGE. Two separate clones (#2 and #4) of two different *B. burgdorferi* background strains (B31-5A4NP1 and B31-5A18NP1 (Kawabata et al. 2004) transformed with *vsp1* or *vsp2* expression plasmids were analyzed.

Next we compared the association of metabolically-labeled wild type or Vsp-recombinant *B. burgdorferi* B313 by directly measuring the radioactivity associated with HBMEC monolayers 4 h after inoculation into the upper transwell chambers. The results showed that both Vsp1 and Vsp2-recombinants bound to the HBMEC monolayers significantly more than the non-recombinant parent clone (Figure 2A). Since B313 is a non-infectious high passage strain that lacks linear and circular plasmids encoding important outer membrane proteins possibly involved in the interaction of *B. burgdorferi* with eukaryotic cells (Table 1), we wanted to confirm these results using Vsp's expressed in low passage, non-infectious background. For this we generated Vsp-expressing recombinant *B. burgdorferi* cells in the B31-5ANP1 background using two different clones, B31-5A18NP1 and B31-5A4NP1 that only lack plasmids lp28-4 and lp56 [14] (Table 1). The *vsp1* and *vsp2* genes were introduced using pBSV2.2, a modified pBSV2 shuttle vector containing a gentamicin resistance marker. Identical to the high passage B313 recombinants [11], the expression of Vsp1 and Vsp2 was driven by the constitutive flagellar promoter *P_flaB_*. Western immunoblots with monoclonal antibodies to Vsp1 and Vsp2 confirmed expression of the respective Vsp protein in all clones tested (Figure 3). The recombinant Vsp's were shown to be surface exposed based on their susceptibility to treatment with proteinase K (Figure 3). Vsp-expressing transformants lost only cp9 and cp32-8 (Table 1) and retained infectivity for SCID mice (not shown). Radiolabeled wild type and Vsp-transformant clones of B31-5A18NP1 were compared for their ability to associate with HBMEC monolayers using the transwell assay as before. The results again showed that the Vsp-transformants associated with HBMEC much more than the wild type (Figure 2B). The association with HBMEC was lower in the low passage, infectious B31-5A18NP1 background compared to the high passage, non-infectious B313 background (Figure 2). We concluded that expression of either Vsp1 or Vsp2 increased the association of *B. burgdorferi* with HBMEC to similar degree and that the infectious background strain showed half the association with HBMEC than the non-infectious background strain.

**Table 1:** Strains used in this study.

Name	Description	Mouse infectivity	Missing plasmids^a^	References
B313	B313 pBSV2	Non-infectious	cp9, cp32-4 to -9, lp21, lp25, lp28-1 to -4, lp36, lp38, lp54, lp56	[11]
B313-Vsp1	B313 pBSV2.vsp1	Non-infectious	cp9, cp32-4 to -9, lp21, lp25, lp28-1 to -4, lp36, lp38, lp54, lp56	[11]
B313-Vsp2	B313 pBSV2.vsp2	Non-infectious	cp9, cp32-4 to -9, lp21, lp25, lp28-1 to -4, lp36, lp38, lp54, lp56	[11]
B31-5A18NP1	ΔBBE02	Infectious	lp28-4, lp56	[14]
B31-5A18NP1-Vsp1	B31-5A18NP1 pCRW1 clone #4	Infectious	cp9, cp32-8, lp28-4, lp56	This study
B31-5A18NP1-Vsp2	B31-5A18NP1 pCRW2 clone #4	Infectious	cp9, cp32-8, lp28-4, lp56	This study

a Plasmids lost in transformants are underlined.

### Strength of the association of Vsp-transformants of *B. burgdorferi* with HBMEC

**Figure 4:**
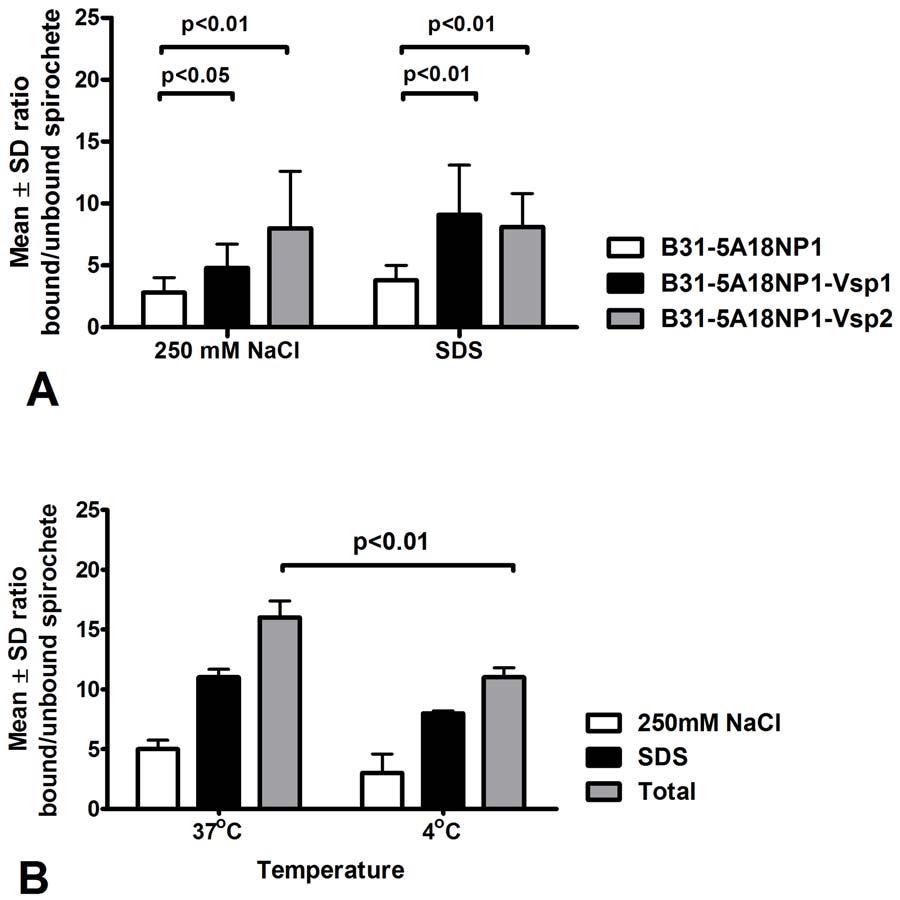
Stringency of the association of Vsp-recombinant *B. burgdorferi* clones with human brain endothelium. HBMEC monolayers on transwell chambers that had been incubated with S35-labeled Vsp-recombinant clones of *B. burgdorferi* at 37°C and rinsed with media were washed with 250 mM NaCl PBS followed by lysis with 0.5% SDS in water (panel A). In some experiments we incubated B31-5A18NP1-Vsp1 with HBMEC also at 4°C (panel B). Results shown the mean ± SD ratio of counts per minute (cpm) associated with the HBMEC monolayer relative to the original inoculum (×100) and represent at least two separate experiments using 8 transwell chambers per clone. Notice that both 250 mM NaCl and 0.1% SDS in water released significantly more radioactivity from HBMEC that had been incubated with the Vsp-recombinant clones. Also notice that the B31-5A18NP1-Vsp1 radioactivity released from HBMEC monolayers by 250 mM NaCl followed by 0.1% SDS decreased by incubation in the cold (panel B).

Next we investigated the strength of the association of Vsp-expressing transformants of B31-5A18NP1 with HBMEC. For this metabolically labeled motile spirochetes were added to confluent HBMEC monolayers in 96 well plates for 4 h at 37°C. The supernatant was collected to measure unbound radioactivity while the monolayers were gently rinsed in media. To measure the strength of the association we first incubated the HBMEC monolayers with 250 nM NaCl in PBS for 10 minutes and harvested the supernatant to measure its radioactivity. Next we incubated the HBMEC monolayers with 0.5% SDS in ddH_2_0 for 10 minutes and harvested the resultant cell lysates to also measure its radioactivity. The data was analyzed as ratios of bound to unbound radioactivity in the 250 nM NaCl wash and in the lysate. The results showed that both the 250 nM NaCl wash and the lysis released radioactivity to a higher degree with the Vsp-transformants than with the wild type strain (Figure 4A). We also examined the effect of incubation at lower temperature on the association of metabolically labeled B31-5A18NP1-Vsp1 with HBMEC. The results showed that the observed association was significantly lowered by incubation at 4°C compared to 37°C (Figure 4B).

### Vsp1 antagonism does not interfere with the association of Bt1 with HBMEC

**Figure 5:**
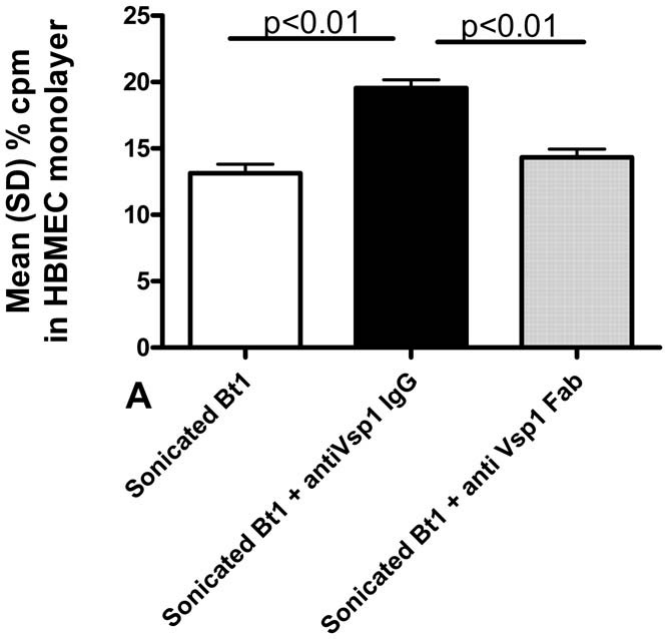
Vsp1 antagonism does not block the association of radioactive proteins of *B. turicatae* serotype 1 (Bt1) with brain endothelium. HBMEC monolayers on transwell chambers were incubated for 20 min with sonicated S35-radiolabeled serotype 1 of *B. turicatae* that had been incubation for 1 h with or without 1 mg/ml of anti Vsp1 IgG3 monoclonal antibody 1H12 or its Fab fragment. Notice that while pre-incubation of Bt1 with the intact monoclonal antibody increased the association of radiolabeled Bt1 proteins to HBMEC, the Fab fragment had no effect.

Since we had been studying the association of Vsp-transformants with HBMEC monolayers by measuring the signal from S35-radiolabeled aminoacids, it was possible that the observed association corresponded to the binding and/intracellular movement of proteins rather than whole spirochetes. To begin investigating this possibility we incubated HBMEC monolayers in transwell chambers with metabolically labeled Bt1 spirochetes that had been sonicated to release proteins into solution. The results showed that sonication did not prevent the association of radioactive protein signal with HBMEC (first column in Figure 5). Since Vsp1 is the most abundant protein of Bt1 spirochetes [5], [15], [16], next we studied whether Vsp1 antagonism may interfere with the observed association. For this we incubated sonicated S35-radiolabeled Bt1 spirochetes with HBMEC monolayers with or without previous pre-incubation for 1 h with 1 mg/ml of IgG3 monoclonal antibody 1H12 that binds to the membrane-distal variable regions 1 and 3 of the Vsp1 dome (O. Kumru and W. Zückert, unpublished results). We also included the Fab fragment of 1H12 to control for possible effects of the Fc fragment of 1H12 on the association [17]. The results showed that pre-incubation with either the whole IgG or the Fab fragment of 1H12 did not reduce the association of sonicated Bt1 proteins with HBMEC (Figure 5). Compared to the Fab fragment, pre-incubation with whole 1H12 IgG increased the association of sonicated Bt1 proteins with HBMEC, suggesting Fc-mediated increased association of Vsp1 with HBMEC.

### Association of Vsp1 with HBMEC

Next we studied whether Vsp1 by itself is capable of associating with HBMEC. For this it was necessary to first isolate, purify, and label Vsp1. Wild type lipidated Vsp1 (LVsp1) was isolated from cultured Bt1 spirochetes by OGP detergent extraction. We similarly purified wild type lipidated Vsp2 (LVsp2) from cultured Bt2 spirochetes since the Vsp2 dome is so different from the Vsp1 dome [18]. To investigate the possible role of the lipidation we used a recombinant non-lipidated Vsp1 that had been previously prepared in *E. coli* [11]. Mouse albumin, a protein abundant in the blood was selected as the non-specific control. The purity of the protein preparations was examined by Coomassie blue staining SDS-PAGE (Figure S1). To label the proteins we selected the lanthanide Europium (Eu)-DTTA, a time-resolved fluorescence (TRF) tag that exhibits high sensitivity and stability when bound to proteins [1], [19], [20]. Extensive testing demonstrated the purity and stability of the Eu-labeled proteins (see methods and Figures S1 and S2).

To investigate whether LVsp1 does associate with HBMEC first we incubated HBMEC in suspension in Eppendorf tubes with Eu-labeled lipidated Vsp1 (Eu-LVsp1) at different concentrations for various times. Non-lipidated Eu-rVsp1 and Eu-albumin were included as controls. A control experiment in which Eu-LVsp1 was added to Eppendorf tubes without HBMEC showed that the observed association of Eu-LVsp1 with HBMEC was not simply the result of Eu-LVsp1 binding non-specifically to the Eppendorf tube (Figure S3).

**Figure 6:**
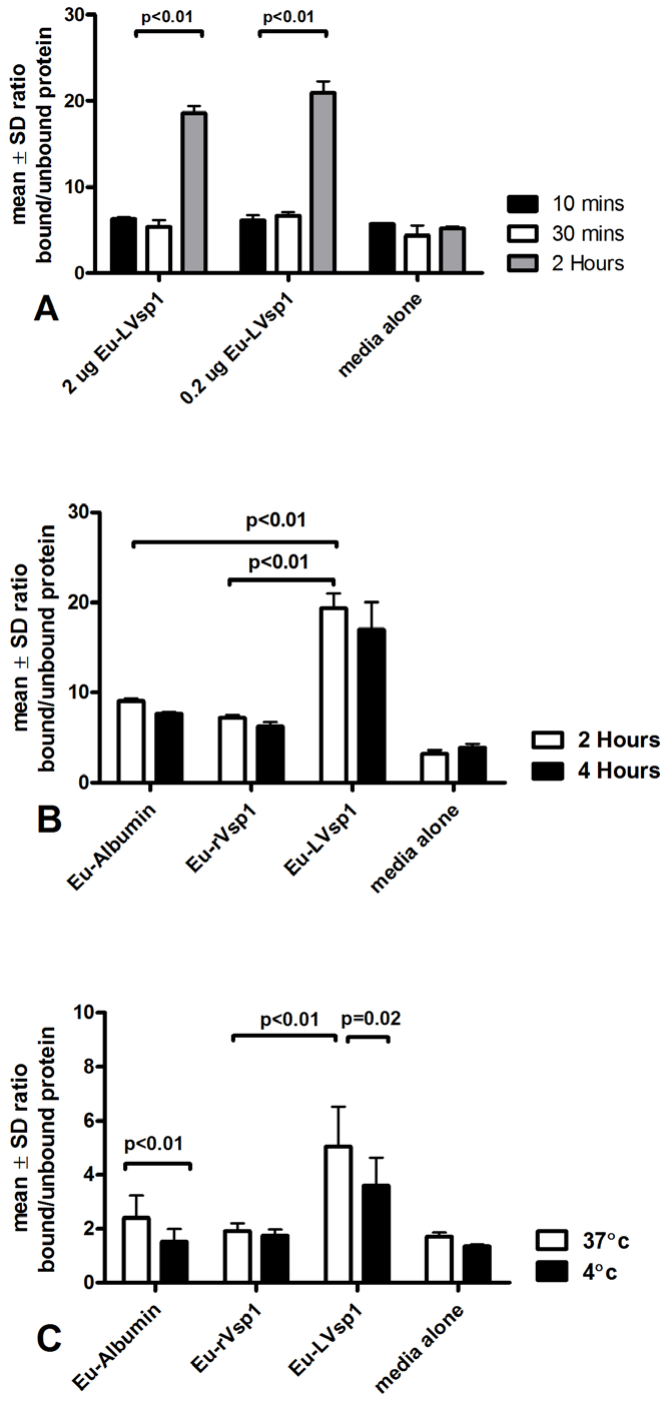
Vsp1 associates with brain endothelium. About 5.5×10^5^ HBMEC in suspension in Eppendorf tubes under gentle rotation were incubated with 0.2 µg of Europium-labeled proteins (unless otherwise indicated) or serum free medium as background control. After incubation the cells were pelleted by centrifugation, washed in HBMEC clear media and the supernatant removed to measure unbound Europium. The cell pellet was lysed in enhancing solution alone (Panels A&B) or enhancing solution followed by lysis in Triton X-100 (panel C only) to recover Eu-labeled proteins associated with the HBMEC. Results are expressed as the mean ± SD ratio of counts per second (cps) of Eu-bound to HBMEC relative to unbound Eu and represent data from at least two separate experiments using 4–6 tubes per protein. Notice that Eu-LVsp1 associated with HBMEC in a time-dependent manner (panels A–C); also notice that the association of LVsp1 with HBMEC was saturable (panel A), did not occur with non-lipidated rVsp1 or albumin (panel B), and decreased by incubation at lower temperature (panel C).

All experiments used 2 h incubation at 37°C under gentle rotation with 0.2 µg of each Eu-labeled protein except were indicated. Following incubation, HBMEC were collected by centrifugation and the supernatant removed for examination by TRF. After rinsing in clear media, the HBMEC pellet was lysed by incubation with enhancing solution followed by sonication and also examined for TRF. Results were expressed as ratio of counts per second (cps) of Eu in the lysate relative to Eu in the unbound supernatant. The results revealed that Eu-LVsp1 associated with HBMEC in a time dependent manner (Figure 6A&B). They also showed that the association was saturable since increasing the amount of input Eu-LVsp1 by 10 fold, from 0.2 to 2 µg, did not result in higher association (Figure 6A). In contrast, increasing the amount of HBMEC did increase the association (not shown). The lipid modification was required for the association since the association of Eu-rVsp1 was similar to that of Eu-albumin and media alone and much lower than that of Eu-LVsp1 (Figures 6B&C). Lowering the incubation temperature to 4°C significantly interfered with the association of Eu-LVsp1 with HBMEC (Figure 6C).

### Vsp1 and Vsp2 interact differently with HBMEC

**Figure 7:**
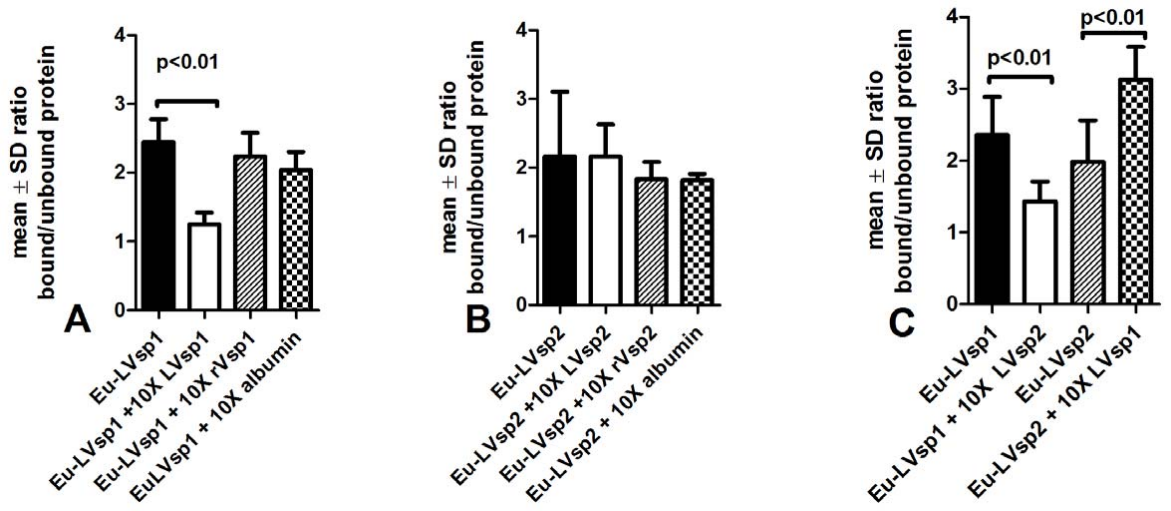
Lipidated Vsp1 or Vsp2 inhibit the association of LVsp1 with brain endothelium non-specifically. 1×10^5^ HBMEC in suspension in Eppendorf tubes were incubated with 0.2 µg of Eu-LVsp1 or Eu-LVsp2 for 2 h at 37°C in the presence or absence of 10-fold excess unlabeled homologous or heterologous Vsp protein. Results are expressed as the mean ± SD ratio of counts per second (cps) of Eu associated to HBMEC relative to unbound Eu and represent data from at least two separate experiments using 8 wells per protein. Notice that the association of Eu-LVsp1 with HBMEC was blocked by 10-fold excess unlabeled LVsp1 (panel A) or LVsp2 (panel C) but not by 10-fold excess unlabeled rVsp1 or albumin (panel A). This did not occur for Eu-LVsp2 (Panel B). Also notice that excess unlabeled LVsp1 increased the association of Eu-LVsp2 with HBMEC (panel C).

Next we compared the association of Vsp1 and Vsp2 with HBMEC. For this we used the same Eppendorf cell suspension assay as before. The results showed that both proteins showed similar association with HBMEC: the mean ± SD ratios of bound to unbound cps were 2.16±(0.93) for Eu-LVsp1 and 2.7±(0.4) for Eu-LVsp2 (p = 0.30, Figure 7A&B). We also examined the mechanism of association of LVsp1 and LVsp2 with HBMEC using protein competition experiments. First we incubated Eu-LVsp1 with HBMEC in the presence of 10-fold higher concentrations of unlabeled LVsp1, rVsp1, or albumin. The results showed that only excess unlabeled LVsp1 interfered with the association (Figure 7A). Next we did the same with Eu-LVsp2 by incubating it with 10 times excess unlabeled LVsp2, rVsp2, and albumin. Unexpectedly, the results showed that none of them interfered with the association (Figure 7B). Therefore, we proceeded to study the degree to which excess unlabeled heterologous Vsp proteins interfered with the association each of the two Vsp proteins with HBMEC. The results showed that 10-fold excess unlabeled LVsp2 interfered with the association of Eu-LVsp1 with HBMEC but that 10-fold excess unlabeled LVsp1 had the opposite effect: it increased the association of LVsp2 with HBMEC (Figure 7C).

### LVsp1 enhances the movement of LVsp2 across the blood-brain barrier

**Figure 8:**
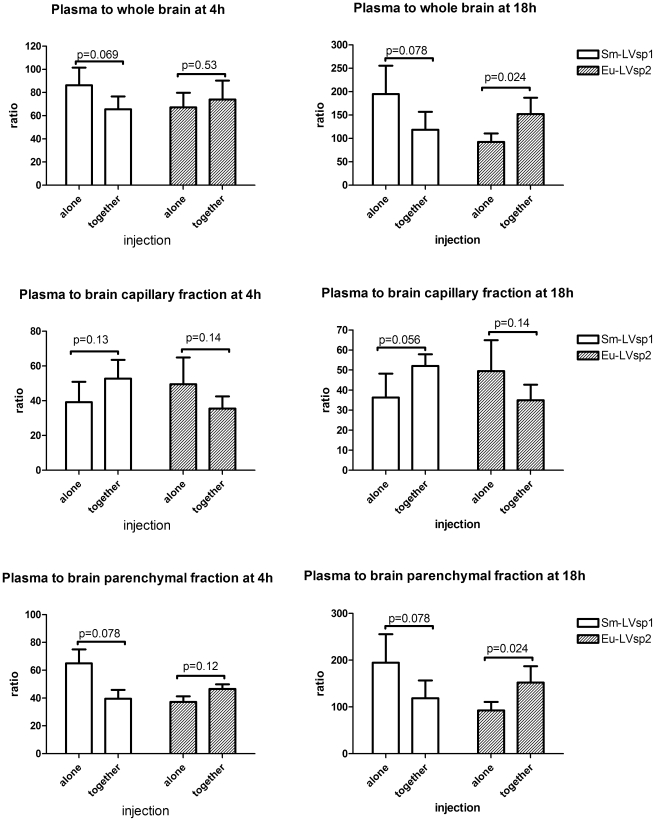
LVsp1 increases the movement of LVsp2 from blood to brain parenchyma in mice. The movement of Samarium Eu-LVsp1 (Sm-LVsp1) and Eu-LVsp2 from blood to brain was studied in groups of 5–6 C57BL10 mice 4 h or 18 h after intraperitoneal inoculation with 100 µg/kg of each protein alone or together. Results were calculated for whole brain (upper panels), capillary enriched brain fraction (middle panels), or capillary-depleted brain parenchymal fraction (lower panels). Results are presented as mean ± SD ratio of counts per second for Sm-LVsp1 or Eu-LVsp2 per microgram of protein extract from plasma or brain tissue. Notice that the movement of Sm-LVsp1 and Eu-LVsp2 from blood to brain increased between 4 and 18 h and that Sm-LVsp1 when given alone moved from blood to brain more than Eu-LVsp2, as recently reported [1]. The co-administration of Eu-LVsp1 with Sm-LVsp1 increased its distribution from blood to the brain parenchymal fraction.

Since LVsp1 had increased the association of LVsp2 with HBMEC next we investigated whether the presence of LVsp1 may also increase the movement of LVsp2 across the blood-brain barrier. For this we used mice that had been injected intraperitoneally with 100 µg/kg of Sm-LVsp1 and 100 µg/kg of Eu-LVsp2 alone or in combination and necropsied 4 h or 18 hours later [1]. The movement of Eu-LVsp2 from blood to brain was examined using ratios that compare the counts per second per microgram of protein of whole brain or brain fraction (capillary depleted or capillary-enriched) relative to plasma as before [1]. The results showed that the presence of Sm-L-Vsp1 increased the dissemination of Eu-LVsp2 from blood to the brain parenchymal fraction in a time dependent manner (Figure 8). We concluded that LVsp1 enhances the movement of LVsp2 across the blood-brain barrier.

## Discussion

In a previous study we have shown that the RF spirochete Bt1 preferentially associates with HBMEC over fibroblasts or arachnoidal human cells; this strong association of Bt1 with HBMEC was shared by isogenic serotypes Bt2 and Bt3 that differ only in the Vsp protein they express [10]. The same study had suggested that the observed association could represent the interaction of Vsp1 –rather than Bt1- with HBMEC as evidenced by the fact that it did not decrease when Bt1 was heat-killed prior to inoculation into the upper transwell chamber [10]. In the present study we expanded the study of the interaction of Vsp1 and Vsp2 with brain endothelium using a variety of approaches, including: (1) metabolically labeled Vsp1 and Vsp2 expressing *B. burgdorferi* transformants; (2) radiolabeled sonicated Bt1 proteins; (3) lanthanide-labeled lipidated and non-lipidated Vsp1 and Vsp2. All experiments in vitro used HBMEC, a human brain endothelial cell line that has many characteristics of blood-brain barrier endothelial cells and has proven useful in previous studies with diverse pathogens, including *Escherichia coli* [21], [22], *B. burgdorferi* [23], and *B. turicatae* [10]. The in vivo studies used mice that had been injected with lanthanide-labeled proteins as part of a previous experiment. The major findings of the present study are the following: (i) Heterologous expression of Vsp1 or Vsp2 increase the association of *B. burgdorferi* with HBMEC to similar degree. (ii) The high passage, non-infectious B313 strain of *B. burgdorferi* associates with HBMEC more than the low passage infectious B31-5A18NP1 strain. (iii) Eu-LVsp1 by itself associates with HBMEC in a time dependent manner. (iv) The association of Eu-LVsp1 with HBMEC is saturable, requires the lipidation, decreases at lower temperature, and is reduced to a similar degree by excess unlabeled LVsp1 or LVsp2. (v) Pre-incubation with an antibody specific for the variable dome region of Vsp1 or with excess unlabeled rVsp1 does not interfere with the association of Vsp1 with HBMEC. And (vi) LVsp1 increases the association of LVsp2 with HBMEC in vitro and its movement across the blood-brain barrier in vivo.

The similarly increased association of *B. burgdorferi* cells with HBMEC, independent of whether they express Vsp1 or Vsp2, indicates that these two members of the Vsp/OspC family of *Borrelia* outer membrane lipoproteins [11], [24]–[26] may be brain endothelial cell adhesins. However, an alternative explanation is that the increased radioactivity associated with the HBMEC monolayers is due to increased binding of metabolically labeled proteins released from the spirochetes rather than due to binding or cell association of the spirochetes themselves. The findings that Vsp1 and Vsp2 by themselves are capable of associating with HBMEC (Figures 6 and 7) and capable of disseminating from blood to brain in mice ([1] and Figure 8) is supportive of this. It is possible that this property is shared by other members of the Vsp/OspC protein family, since association with HBMEC monolayers has been observed with multiple isogenic serotypes of *B. turicatae* [10], isogenic serotypes of *B. hermsii* [27], and non-Vsp expressing strains of *B. burgdorferi* (Figure 2).

The precise mechanism by which the association of Vsp1 with brain endothelium takes place remains to be determined. Yet, some important clues are provided by putting our current findings in the context of previous findings. First, the observed that the association preferentially occurs with endothelial cells as Vsp1-expressing *B. burgdorferi* transformants like their Bt1 counterpart, associated with HBMEC much more than with fibroblasts or arachnoidal human cells (Figure 1C). Second, the association does not require either live or intact bacteria (Figure 5 and [10]), as it was observed with sonicated proteins from Bt1 and purified Vsp1 and Vsp2. Third, the lipidation is very important for the observed association (Figures 6–7). Fourth, the observed association does not appear to simply represent loose extracellular binding since incubation with higher concentrations of salt and cell lysis released considerable amounts of protein from HBMEC that had been already washed (Figure 4). Fifth, the presence of one or more receptors on the HBMEC membrane is required for the association because pre-treatment of HBMEC with proteinase K completely prevents the association [10]. Finally, the finding that incubation at 4°C lowered the association of both Vsp1-transformants (Figure 4) and Eu-LVsp1 (Figure 6) with HBMEC is consistent with protein membrane traffic rather than simple membrane binding. This is in agreement with the previous observation by immunofluorescence microscopy that Vsp1 released from Bt1 can be seen intracellularly in HBMEC [10]. It is well established that when endothelial cells are incubated with lipoproteins at 4°C, almost all of the receptor-bound ligands remain on the cell surface [28]; this is commonly referred to as “cell binding”. In contrast, when endothelial cells are incubated with lipoproteins at 37°C both the lipoproteins bound to the cell surface and receptor-bound ligands are internalized by the cells [28]; this is referred to as “cell-association”. We therefore propose that the observed association of LVsp1 with HBMEC represents cell association.

It is very clear that the lipidation plays a critical role in the association of LVsp1 with HBMEC, although the same does not appear to be the case for LVsp2 (Figures 6–7). Furthermore, the finding that unlabeled excess LVsp1 or LVsp2 reduced the association of Eu-LVsp1 with HBMEC to a similar degree (Figure 6C) indicates that a specific sequence of the variable dome region is not critical [18]. This is supported by the observation that pre-incubation with the anti Vsp1 monoclonal antibody 1H12 did not interfered with the association (Figure 5). Others have previously shown that *Borrelia* lipoproteins can spontaneously associate with spirochetal membranes and that this is mediated by the lipidation [29].

What is more difficult to explain is the limited ability of LVsp2 to disseminate from blood to the brain parenchyma relative to Vsp1 [1]. The binding of LVsp2 to HBMEC is similar to that of LVsp1 and is not inhibited by LVsp1 (Figure 7). Previous studies have shown that Vsp2 appears to be more “sticky” than Vsp1, as shown by its greater binding to extracellular matrix components like heparin and dermatan sulfate [13]. This same increased “stickiness” to extracellular matrix may explain why the pathogen load in collagenous tissues like the skin and the heart is much greater during persistent infection with Bt2 than with Bt1 [2], [3], [30], [31].

It is intriguing that the presence of LVsp1 increased the association of LVsp2 with HBMEC in vitro (Figure 7) and the movement of LVsp2 from blood to the brain parenchymal fraction in mice (Figure 8). Previous studies have shown that Vsp1 and Vsp2 have protease-resistant, thermostable cores and aggregate as dimers in solution [32]. If Vsp1 and Vsp2 could form heterodimers in solution as has been observed with other bacterial proteins [33], this could explain the enhancing effect that the presence of LVsp1 had on the association of LVsp2 with HBMEC and its traffic across the blood-brain barrier in mice. LVsp2 has the same lipidation but is 32% different at the amino acid level from LVsp1 [16],[34]; all the differences between LVsp1 and LVsp2 concentrate in the variable dome region [11], [18], [32]. Computer modeling of Vsp2 using the crystal structure of Vsp1 has shown that although both protein domes are hydrophilic, the Vsp2 dome has many fewer charged residues. As a result, the dome of Vsp1 is more polar and its negative electrostatic potential is more pronounced than that of Vsp2. Such increased polarity of the Vsp1 dome may facilitate the non-specific binding to electrostatically charged aminoacid residues on the HBMEC membrane leading to better cell internalization via adsorptive endocytosis. An endocytic mechanism has been shown to be used for internalization of *E. coli* into HBMEC [22].

Our knowledge of the proteins important for brain infection by borrelia spirochetes remains rather limited [35]–[37] compared to many other pathogenic bacteria [38]. Several spirochetal proteins that bind to eukaryotic cells and/or tissues have been identified, including members of the Vsp and Vlp family from RF spirochetes [13], [27], and Osp [39], [40], Dbp [41], and VlsE [40] from Lyme disease spirochetes. On the host's side, several molecules have been implicated in the binding of eukaryotic cells and/or the extracellular matrix to spirochetes, including integrins [42], glycolipids [43], proteoglycans [41], [44], and glycosaminoglycans (GAGs) [45], [46]. There have been several previous studies of the interaction of borrelia spirochetes with endothelial cells. They have revealed that borrelias can cross intercellular junctions of human umbilical vein endothelial cells (HUVEC) [47]–[50] and HBMEC monolayers [23]; one study reported that borrelias can even be internalized into endothelial cells [51]. Several authors have shown that borrelias can activate endothelium [50], [52]–[58]. One particular observation which is more relevant to the current study is the demonstration of differential binding of Vsp-expressing borrelias to glycosaminoglycans (GAG) [11]; this suggests that Vsp2-expressing spirochetes might get stuck in extracellular matrix or even on brain endothelium due to their GAG-binding properties, while Vsp1-expressing ‘teflon’ spirochetes evade that fate and ultimately get swept into the brain parenchyma.

The observed phenotypic differences between high and low passage *B. burgdorferi* background clones might be explained by their different surface architectures: During extensive passage, the *B. burgdorferi* strain derivative B313 lost the majority of its plasmids, and with it the ability to express many of the strains' plasmid-encoded surface proteins such as OspA [32], [39]. In this background, ectopically expressed Vsp proteins are prominently displayed on the surface without much interference from other proteins, therefore leading to a pronounced change in phenotype. In contrast, the low passage *B. burgdorferi* B31 clone B31-5A18NP1 [14] exhibits a much more complex surface, where multiple abundant proteins such as Osp's might at least partially mask the expressed Vsp proteins and therefore lead to a somewhat attenuated change in phenotype.

Further work is necessary to characterize the precise mechanism underlying the observed association of Vsp1 with HBMEC. Current limitations in deciphering the role of surface lipoproteins from relapsing fever borrelias in host-pathogen interactions will also need to be addressed. Studies with primary human brain endothelial cells might reveal differences to the immortalized HBMEC cell lines. To conclusively satisfy “Koch's molecular postulates” for the biological role of Vsp's, genetic tools for the manipulation of *B. turicatae* need to be developed.

## Materials and Methods

### Bacterial strains, plasmids, culture, and mouse infectivity

Serotype 1 of *B. turicatae* has been previously described [5], [15]. Since *B. turicatae* is currently not amenable to genetic manipulation this precluded us from preparing *vsp* deletion, i.e. loss-of-function mutants of *B. turicatae*. As an alternative we engineered the Lyme disease spirochete *B. burgdorferi* to express Vsp1 or Vsp2 in the outer membrane for gain-of-function experiments. This approach was used previously to demonstrate the effects of Vsp on the interaction of spirochetes with glycosaminoglycans [11], [13]. All recombinant *B. burgdorferi* strains used are cloned derivatives of the type strain B31 (ATCC 35210). The high-passage non-infectious B313-based strains were described earlier [11]. In these strains, *vsp1* and *vsp2* are encoded on recombinant pBSV2-derived plasmids [59] and their expression is driven by the constitutive *B. burgdorferi flaB* promoter P*_flaB_*. The low-passage *B. burgdorferi* strains were based on infectious clones B31-5A4NP1 and B31-5A18NP1 [14]. To introduce *vsp1* and *vsp2* into these two kanamycin-resistant background strains, we first modified pBSV2 by replacing its kanamycin (*aphI*) resistance marker with a gentamicin resistance (*aacC1*) marker. The *aacC1* gene was amplified from pKK82 [60] by PCR using thermostable proofreading Pfx DNA polymerase (Invitrogen, Carlsbad, Calif.) and primers NdeaacC1-fwd (5′- GGAATTCCATATGTTACGCAGCAGCAACGA-3′) and NcoAataacC1-rev (5′-CATGCCATGGGACGTCTTAGGTGGCGGTAC-3′). The amplicon was digested with *Nde*I and *Aat*II and ligated to an *Nde*I-*Aat*II pBSV2 backbone fragment, resulting in pBSV2.2. Next, *Bam*HI-*Hin*dIII fragments containing the P*_flaB_*-*vsp* expression cassettes were excised from pBSV2.*vsp1* and pBSV2.*vsp2*, respectively [11], and ligated to a *Bam*HI-*Hin*dIII-pBSV2.2 backbone fragment, resulting in plasmids pCRW1 (harboring *vsp1*) and pCRW2 (harboring *vsp2*). B31-5A18NP1 cells were transformed with pCRW1 and pCRW2 by electroporation as described [61]. Transformants were selected on BSK-II plates containing 200 µg/ml kanamycin and 40 µg/ml gentamicin and multiple independent clones each were expanded in selective liquid BSK-II [11]. *B. burgdorferi* plasmid profiles of background strains and transformants were established by PCR using plasmid-specific oligonucleotide primers [62], [63]. All clones retained plasmids lp25 and lp28-1, which are essential for infectivity. Protein lysates from all clones were examined by Western blot for expression of Vsp 1, Vsp2, OspA, and FlaB with monoclonal antibodies 1H12, 5F12, H5332, and H9724, respectively [15], [64]. The only plasmids lost in some or all of the transformants were cp9, cp32-8, lp21, lp28-4 and lp56. One clone each was used for further studies (Table 1). Infectivity was tested by culturing skin samples from groups of 6–8 mice with severe combined immunodeficiency (SCID) 2 weeks after intradermal inoculation with 10^4^ *B. burgdorferi* spirochetes per mouse. *Borrelias* were cultured at 34°C in BSK-H medium containing 6% rabbit serum (Sigma) and counted in a Petroff-Hausser chamber under phase-contrast microscopy. Recombinant expression of Vsp1 or Vsp2 was assessed by Western blot with serotype-specific monoclonal antibodies and rabbit hyperimmune sera against strain N40 of *B. burgdorferi* as positive control. For cell incubation experiments, the bacteria were grown in medium supplemented with 50 mCi ml^−1^ (^35^S)-methionine and (^35^S)-cysteine (Amersham) as before [10]. At late log phase, cells were pelleted by centrifugation at 13,000 *g* for 20 minutes at room temperature, washed with BSK-H three times to remove unbound radioactivity, resuspended in fresh BSK-H media with 10% DMSO at a concentration of 10^8^ spirochetes/ml, aliquoted, and stored frozen at −80°C to be used within two months. Before use, 20% tri-chloroacetic acid was used to precipitate radiolabeled proteins from the frozen stocks; we confirmed that ≥80% of the radioactivity was protein-bound. Radiolabeled spirochetes were examined microscopically after thawing to confirm their viability and only used if >80% were motile.

### Eukaryotic cell cultures

HBMEC [21], [22], SV40-transformed IMR90 human fibroblasts [65], and F5 human meningioma/arachnoidal cells [66] were grown at 37°C in a 5% CO_2_ atmosphere in media containing 10% fetal bovine serum. HBMEC media was RPMI1640, 10% NuSerum, MEM vitamins, MEM non-essential amino acids, 1 mM sodium pyruvate, 2 mM L-glutamine, 30 µg/ml endothelial growth supplement, and 5 U/ml heparin [21], [22]. IMR90 media was 1∶1 DME/F10; F5 media was RPMI. Preliminary experiments showed that *B. burgdorferi* remained viable in HBMEC, IMR90, and F5 media for up to 24 h while none of the eukaryotic cell lines grew well in BSK media. Cells were released from culture flasks by trypsinization, resuspended in fresh medium, and counted under phase contrast microscopy with a hemocytometer after mixing with trypan blue to examine viability.

### Transwell chamber assays

Two sizes of collagen-coated, 3.0 µm pore membranes for transwell chambers were used, 12 mm diameter membrane inserts (Corning Costar 3494) placed in 12-well culture plates (Corning Costar 3513) for multiple time point sampling and 6.5 mm diameter membrane inserts (Corning Costar 3496) placed in 24-well culture plates (Corning Costar 3526) for single time point sampling. For both transwells 5×10^5^ eukaryotic cells were seeded in the upper chambers and allowed to grow to confluency overnight. For the 12 mm transwells the volumes were 500–600 µl in the upper chamber and 1,000-1,500 µl in the lower chamber; 20 µl were removed at each time point from both chambers without replacing the volume. For the 6.5 mm transwells the volumes were 100 µl in the upper chamber and 600 µl in the lower chamber. HBMEC were grown to confluency overnight. Monolayer confluency was examined by adding 50 mg of 2,000 dextran blue (Sigma) in DMEM clear media to the upper transwell chamber and measuring the optical density in aliquots removed from the upper and lower chamber by spectrophotometry after 4 h. For experiments with metabolically labeled spirochetes ∼5×10^4^ counts per minute (cpm) of S35-aminoacid labeled borrelial stocks, corresponding to ∼1×10^7^ spirochetes total, were added to the upper chamber. In the multiple time point experiments the radioactivity associated with the monolayer was measured indirectly using the counts of the original inoculum and individual 20 µL aliquots from the upper and lower chambers. In the single time point experiments it was measured directly by removing the monolayer after rinsing and drying and placing it into liquid scintillation cocktail (Ecolite). In some experiments S35-radiolabeled proteins from sonicated Bt1 spirochetes were pre-incubated for 1 hour at 34°C with ∼1 mg/ml of anti Vsp1 IgG3 mouse monoclonal antibody 1H12 [15], its Fab fragments of 1H12 (Pierce Cat# 44985), or media alone as a control prior to inoculation into the upper transwell chamber. 1H12 binds specifically to the distal variable region of Vsp1 (W. Zückert, personal communication).

### Protein preparation and labeling

Lipidated Vsp1 (LVsp1) and Vsp2 (LVsp2) were prepared from BSK cultures of serotype 1 (Bt1) or 2 (Bt2) of *B. turicatae* by extraction with Octyl β-D-glucopyranoside (OGP) as described [15]. Both Vsp1 and Vsp2 preparations caused significantly increased serum levels of IL-10 upon intraperitoneal injection into mice at 100 µg/kg (not shown), as expected for bacterial lipoproteins [67]. Further evidence that purified LVsp1 and LVsp2 were lipidated was that their precipitation in solutions at concentrations of OGP ≤0.01%. Mouse albumin was purchased from Sigma (Cat. No. A1056). Recombinant Vsp1 (rVsp1) and Vsp2 (rVsp2) are S34-amino terminally truncated non-lipidated proteins made in *Escherichia coli* by cloning the *vsp1* or *vsp2* genes of B1 or Bt2, respectively [11]. Protein concentration was measured using the BCA^TM^ Protein Assay Kit (Pierce Cat No. 23227). Protein purity was verified by SDS-PAGE (Figure S1A) and their identification by Western blot with anti Vsp1 (1H12) or Vsp2 (5F12) monoclonal antibodies [15], [34] (not shown). Europium (Eu) labeling of proteins and their purification was performed according to the manufacturer instructions (Perkin-Elmer, Boston MA) as described [1]. Briefly, 1 mg of each protein in 50 mM NaCO3 pH 9.3 was incubated with Europium (Eu) or Samarium (Sm) labeling reagents (DELFIA labeling Kits) at 25°C in the dark for 12 hours. Sephadex G-25 chromatography (Sigma 54805) was used for separation of Eu-labeled protein from free Eu (Figure S1B). After chromatography the proteins were dialyzed using Slide-A-Lyzer dialysis Cassette (Pierce Cat No. 66380) against distilled water at 4°C for 48 hours for further purification (Figure S1C). After dialysis the Eu-labeled proteins were aliquoted and stored at −20°C. Trace OGP (≤0.01%) was added to the lipidated proteins to prevent them from precipitating. Immediately before using them, the proteins were thawed and vortexed for 1 to 2 minutes at 10,000 rpm to bring them into solution.

### Stability of Eu-labeled proteins

To verify the stability of the proteins and the lanthanide labeling we examined aliquots from each of the labeled proteins with the BCA assay kit (Pierce, Product No. 23337) to measure protein concentration and with time resolved fluorescence (TRF) to measure counts per second (cps) after 0, 6, 12 and 24 months of continuous storage at −20°C. We confirmed that both the proteins themselves and the lanthanide-counts were stable for up to 2 years of continuous storage in the cold (Table S1). We also confirmed the stability of the Eu-protein binding by measuring the percentage of Eu-bound to each protein before and after centrifugation on 8,000 MW centrifugal devices (Amicon Ultra15, #UFC901008, Millipore, Bedford, MA) that remove free Eu because of its low molecular weight (<700 dalton) (Figure S2A). We also verified that the TRF signal was coming from Eu-bound to protein rather than from free Europium using precipitation with trichloroacetic acid (TCA) (Figure S2B). For this, an equal volume of ice-cold 5% TCA was mixed with aliquots of 2 µg of each Eu-labeled protein diluted in 200 µl of distillated water followed by centrifugation at 3,000 g for 10 minutes, removal of the supernatant, and resuspension of the pellet in Delfia enhancing solution (Perkin-Elmer) by vortexing. Protein-bound Eu was determined by comparing the cps in the supernatant and the pellet. We confirmed that the signal was coming from Eu-bound to protein rather than free Eu by measuring the cps in protein elutes from bands sectioned from Coomassie-stained 12% SDS-PAGE gels that had been loaded with aliquots of Eu-labeled proteins. For this, we excised bands of the predicted size from the acrylamide gel with a clean razor blade, minced them into smaller pieces, placed them in a Nanosep centrifugal device with a 10 kDa filter in elution buffer, used centrifugation at 3,000 g for 20 minutes to remove free Eu, and measured protein-bound Eu by TRF in aliquots removed from the upper filter chamber.

### Vsp1-HBMEC binding assays

To compare the association of LVsp1 and LVsp2 with HBMEC we used an assay in which HBMEC were incubated with the proteins while in suspension on Eppendorf tubes. For this, 5.5×10^5^ HBMEC suspended in 0.5 ml of serum free clear HBMEC medium in 1.5 ml Eppendorf tubes were kept under gentle rotation and incubated from 10 min to 4 h at 37°C (4°C for some experiments) with 0.2 (2 µg for some experiments) of Eu-labeled proteins alone or together with 10 times excess (2 µg) unlabeled proteins. We verified that the tube rotation did not affect HBMEC viability for up to 2 h using Trypan blue exclusion (not shown) and that the proteins did not bind non-specifically to the walls of the Eppendorf tube (Figure S3). After incubation, HBMEC were harvested by centrifugation at 2,500 rpm (300 *g*) for 10 minutes and the supernatant examined for unbound protein by TRF by mixing with Delphia enhancing solution at 1∶20 ratio. The HBMEC pellet was washed 3 times by gentle resuspension in clear HBMEC medium followed by centrifugation at 2,500 rpm, lysed by resuspension in 200 µL of Delphia enhancing solution (Perkin-Elmer) at room temperature for 1 h (which also releases the Eu-tag from any protein that was associated with the cells), ultrasonication in ice for 20 sec, and centrifugation to pellet cell debris for 25 min in the cold at 13,000 rpm. In some initial experiments an additional lysis in detergent with Triton X-100 was also used (Figure 7C). Results were expressed as mean ± SD ratio of cps bound to unbound to HBMEC after adjusting for the differences in input volume (500 µL for the unbound supernatant and 200 µL for the bound supernatant).

### Vsp dissemination from blood to brain

Groups of 5–7 week old female C3H/HeJ mice were obtained from Taconic Farms (Germantown, NY) were injected intraperitoneally with 100 µg/kg of samarium (Sm)-labeled LVsp1 or/and Eu-labeled LVsp2 diluted in 200 µl of PBS alone or in combination; mice injected with PBS alone were used as background control. Necropsies were done 4 or 18 hours after protein injection. The movement of Sm-LVsp1 and Eu-LVsp2 from blood to brain was measured as before [1]. After extensive intracardiac with buffer at necropsy, the brain was removed, one hemisphere used for whole brain protein extraction and the other half used for capillary depletion by dextran density centrifugation [1] to differentiate the protein signal present in brain capillaries from that present in the brain parenchyma itself.

### IL-10 ELISA

Levels of IL-10 were determined using monoclonal antibody pairs from BD Pharmingen (San Diego, CA) in mouse plasma 12 h after intraperitoneal inoculation of LVsp1, LVsp2, or as negative control rVsp1; the coating antibody was purified anti-mouse IL-10 (Cat 551215) and the detection antibody was biotinylated rat anti-mouse IL-10 (Cat 554423).

### Statistical analyses

Comparisons of counts per minute (for radioactivity) or counts per second (for TRF), either as absolute numbers, percentage of total counts, or ratios of bound to unbound were done using a one way (Kruskal-Wallis test with Dunn's test for multiple comparisons) or two-way ANOVA (Bonferroni post-test for multiple comparisons). Unpaired t-test were used to compare the movement of proteins from blood to brain/brain fractions when injected alone or in combination. GraphPad Prism software version 5.00 for Windows was used for all calculations (GraphPad Software, San Diego California USA). A *p* value ≤0.05 was considered significant.

## Supporting Information

Figure S1 Purification and lanthanide-labeling of Vsp1 and control proteins. Panel A - Protein preparations were separated on a 12% SDS-PAGE gel; Coomassie-blue staining revealed only one band of the expected size for each protein without any contaminant (lane 2, LVsp1; lane 3, rVsp1, lane 4, albumin). Notice the absence of the 41kDa flagellin band in all protein preparations. The molecular weight of the proteins in kDa is indicated to the left in lane 1. Panel B - Sephadex G-25 gel chromatography of Eu-labeled LVsp1. The first peak represents Eu-LVsp1 and the second peak free Eu-chelate. The solid line illustrates the protein concentration in nmol/L and the dotted line represents Eu concentration in nmol/L. Similar results were obtained for Eu-rVsp1 and Eu-albumin (not shown). Panel C - Time course removal of residual free Eu from Eu-labeled LVsp1 by ddH20 dialysis. The rate of free Eu removal was determined by measuring Eu concentration on each of 8 fractions of exchanged water. The data represents mean ± SD nmol of Eu/L. The dotted line represents background time-resolved fluorescence.
(0.65 MB TIF)

Figure S2 Stability of Europium-labeled proteins. We confirmed the stability of the Eu-protein binding by measuring the percentage of Eu-bound to each protein before and after centrifugation on 8,000 MW centrifugal devices that remove free Eu because of its low molecular weight (<700 dalton) (panel A). We also verified that the TRF signal was coming from Eu-bound to protein rather than from free Europium using precipitation with trichloroacetic acid (TCA) (Panel B).
(0.27 MB TIF)

Figure S3 Lanthanide-labeled proteins do not bind to the Eppendorf tubes. We showed by adding Eu-LVsp1 to Eppendorf tubes with and without HBMEC that there is little binding to the tube itself.
(1.33 MB TIF)

Table S1 Prolonged storage in the cold does not affect the concentration of protein or the time-resolved fluorescence of Europium.
(0.04 MB DOC)
