# Supplementary figures and images for: Interaction of Variable Bacterial Outer Membrane Lipoproteins with Brain Endothelium
Source: PLoS One. 2010 Oct 22;5(10):e13257. doi: 10.1371/journal.pone.0013257 (PMC2962627; doi:10.1371/journal.pone.0013257)

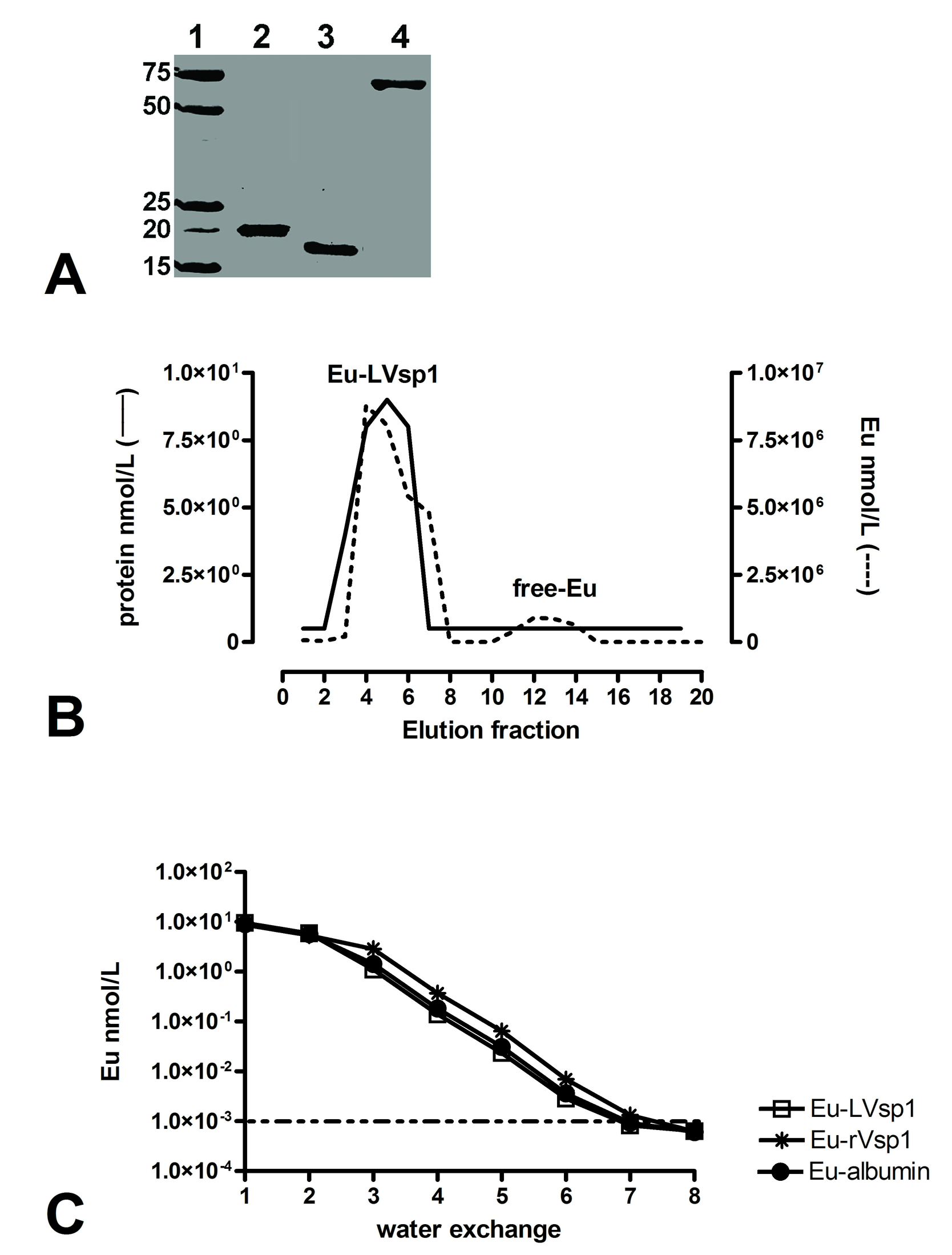

Supplement: Figure S1 — Purification and lanthanide-labeling of Vsp1 and control proteins. Panel A - Protein preparations were separated on a 12% SDS-PAGE gel; Coomassie-blue staining revealed only one band of the expected size for each protein without any contaminant (lane 2, LVsp1; lane 3, rVsp1, lane 4, albumin). Notice the absence of the 41kDa flagellin band in all protein preparations. The molecular weight of the proteins in kDa is indicated to the left in lane 1. Panel B - Sephadex G-25 gel chromatography of Eu-labeled LVsp1. The first peak represents Eu-LVsp1 and the second peak free Eu-chelate. The solid line illustrates the protein concentration in nmol/L and the dotted line represents Eu concentration in nmol/L. Similar results were obtained for Eu-rVsp1 and Eu-albumin (not shown). Panel C - Time course removal of residual free Eu from Eu-labeled LVsp1 by ddH20 dialysis. The rate of free Eu removal was determined by measuring Eu concentration on each of 8 fractions of exchanged water. The data represents mean ± SD nmol of Eu/L. The dotted line represents background time-resolved fluorescence. (0.65 MB TIF) [file pone.0013257.s001.tif]

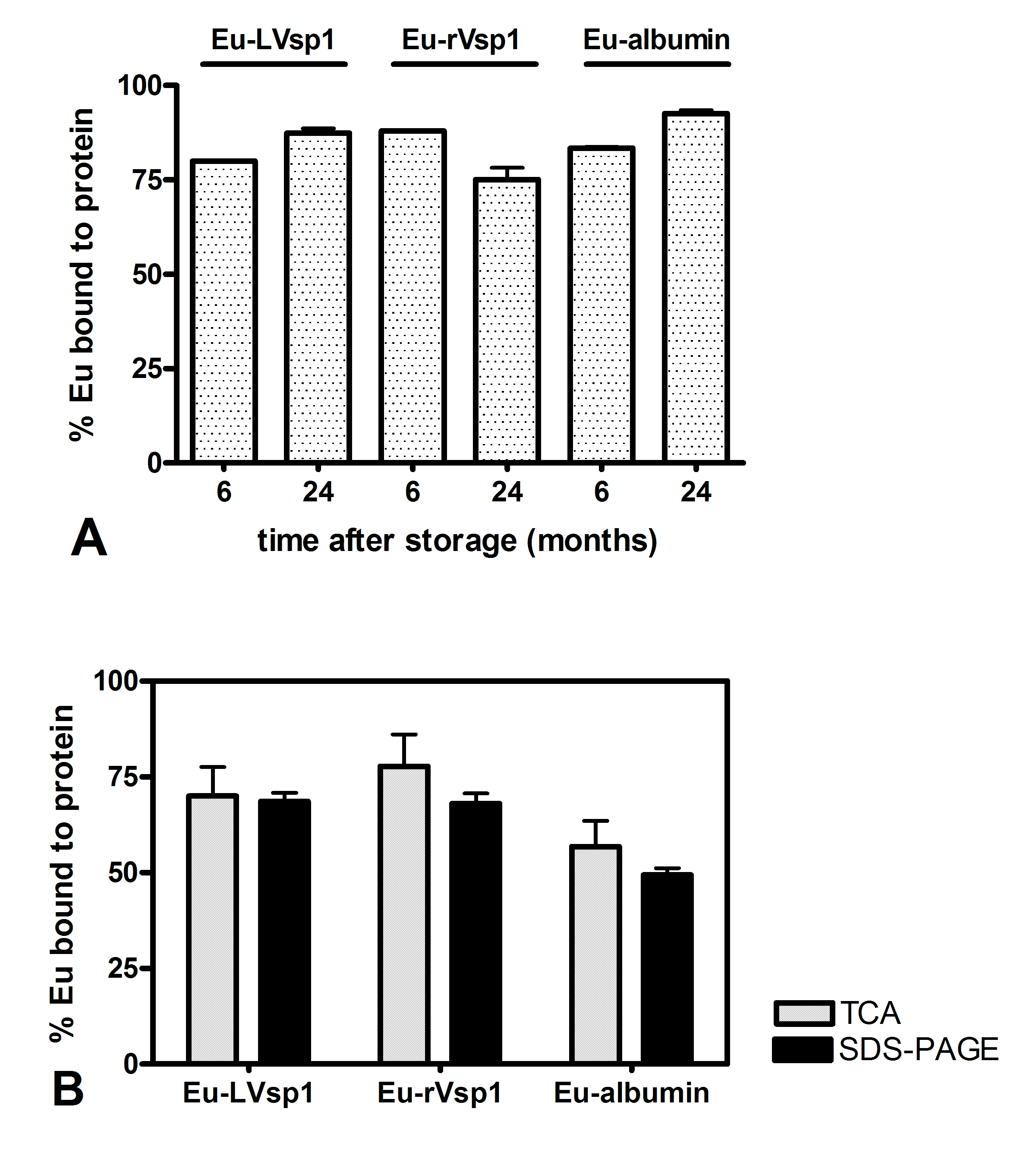

Supplement: Figure S2 — Stability of Europium-labeled proteins. We confirmed the stability of the Eu-protein binding by measuring the percentage of Eu-bound to each protein before and after centrifugation on 8,000 MW centrifugal devices that remove free Eu because of its low molecular weight (<700 dalton) (panel A). We also verified that the TRF signal was coming from Eu-bound to protein rather than from free Europium using precipitation with trichloroacetic acid (TCA) (Panel B). (0.27 MB TIF) [file pone.0013257.s002.tif]
