# Supplementary material for: Interaction of Variable Bacterial Outer Membrane Lipoproteins with Brain Endothelium
Source: PLoS One. 2010 Oct 22;5(10):e13257. doi: 10.1371/journal.pone.0013257 (PMC2962627; doi:10.1371/journal.pone.0013257)
Supplement: Table S1 — Prolonged storage in the cold does not affect the concentration of protein or the time-resolved fluorescence of Europium. (0.04 MB DOC) [file pone.0013257.s004.doc]

| **Supplementary Table 1.** Measurement of protein concentration and Europium fluorescence during storage at -20^o^C over 2 years | | | | | | |
| --- | --- | --- | --- | --- | --- | --- |
| Months | Eu-LVsp1 | | Eu-rVsp1 | | Eu-albumin | |
|  | mg/ml^a^ | Eu fluorescence | mg/ml^a^ | Eu fluorescence | mg/ml^a^ | Eu fluorescence |
| 0 ^b^ | 0.2(0.05) ^c^ | 4.9E+11(3.7E+09) ^d^ | 0.75(0.01) | 9.8E+11(4.2E+10) | 0.2(0.04) | 1.2E+12(4.7E+09) |
| 6 | 0.19(0.03) | 5.5E+11(1.6E+10) | 0.73(0.03) | 8.1E+11(5.1E+09) | 0.2(0.08) | 1.0E+12(1.4E+10) |
| 12 | 0.2(0.09) | 1.6E+10(2.6E+10) | 0.75(0.05) | 9.4E+11(4.9E+10) | 0.19(0.06) | 1.2E+12(2.9E+10) |
| 24 | 0.18(0.07) | 5.1E+11(2.1E+09) | 0.72(0.01) | 1.1E+12(8.2E+10) | 0.2(0.01) | 1.09+12(6.3E+09 |
| ^a^Protein concentration | | | | | | |
| ^b^Time in storage after labeling | | | | | | |
| ^c^Values represent mean (SD) | | | | | | |
| ^d^Counts per second | | | | | | |
